# Electroacupuncture Alleviates Anxiety-Like Behaviors Induced by Chronic Neuropathic Pain via Regulating Different Dopamine Receptors of the Basolateral Amygdala

**DOI:** 10.1007/s12035-022-02911-6

**Published:** 2022-06-13

**Authors:** Mengwei Wu, Yeqing Chen, Zui Shen, Yichen Zhu, Siqi Xiao, Xixiao Zhu, Zemin Wu, Jinggen Liu, Chi Xu, Pingan Yao, Weiwei Xu, Yi Liang, Boyi Liu, Junying Du, Xiaofen He, Boyu Liu, Xiaoming Jin, Jianqiao Fang, Xiaomei Shao

**Affiliations:** 1 Key Laboratory of Acupuncture and Neurology of Zhejiang Province, Department of Neurobiology and Acupuncture Research, The Third Clinical Medical College, Zhejiang Chinese Medical University, No. 548, Binwen Road, Binjiang DistrictZhejiang Province, Hangzhou City, China; 2 Department of Anatomy, Cell Biology and Physiology, Stark Neurosciences Research Institute, Indiana University School of Medicine, Indianapolis, IN USA

**Keywords:** Neuropathic pain, Anxiety, Basolateral amygdala, Dopamine receptors, Electroacupuncture

## Abstract

Chronic pain, such as neuropathic pain, causes anxiety and other negative emotions, which aggravates the pain sensation and increases the risk of chronic pain over time. Dopamine receptor D1 (DRD1) and dopamine receptor D2 (DRD2) in the basolateral amygdala (BLA) have been implicated in mediating anxiety-related behaviors, but their potential roles in the BLA in neuropathic pain-induced anxiety have not been examined. Electroacupuncture (EA) is commonly used to treat chronic pain and emotional disorders, but it is still unclear whether EA plays a role in analgesia and anxiety relief through DRD1 and DRD2 in the BLA. Here, we used western blotting to examine the expression of DRD1 and DRD2 and pharmacological regulation combined with behavioral testing to detect anxiety-like behaviors. We observed that injection of the DRD1 antagonist SCH23390 or the DRD2 agonist quinpirole into the BLA contributed to anxiety-like behaviors in naive mice. EA also activated DRD1 or inhibited DRD2 in the BLA to alleviate anxiety-like behaviors. To further demonstrate the role of DRD1 and DRD2 in the BLA in spared nerve injury (SNI) model-induced anxiety-like behaviors, we injected the DRD1 agonist SKF38393 or the DRD2 antagonist sulpiride into the BLA. We found that both activation of DRD1 and inhibition of DRD2 could alleviate SNI-induced anxiety-like behaviors, and EA had a similar effect of alleviating anxiety. Additionally, neither DRD1 nor DRD2 in the BLA affected SNI-induced mechanical allodynia, but EA did. Overall, our work provides new insights into the mechanisms of neuropathic pain-induced anxiety and a possible explanation for the effect of EA treatment on anxiety caused by chronic pain.

## Introduction

Pain, an unpleasant, subjective feeling, and emotional experience related to tissue injury or potential injury [1, 2], has multiple dimensions, including sensory recognition, emotional motivation, and cognitive evaluation [3]. Chronic pain, such as neuropathic pain, is a major public health problem worldwide. Currently, the number of people with chronic pain accounts for more than 1/5 of the world’s total population [4], and the prevalence of chronic pain in China is as high as 31.54% [5]. Chronic pain may set up an unstable emotional state and induce negative emotions, most commonly, anxiety and depression disorders [6]. These pain emotions, especially anxiety, aggravate pain sensation and increase the risk of chronic pain. An increasing number of studies have reported that pain-induced anxiety results in decreased immune function, impaired decision making, worse impulse control, increased sleep loss [7], and physiological imbalance in eating [8], resulting in prolonged pain that is difficult to cure. Although the effect of chronic neuropathic pain-induced anxiety is very serious, its internal mechanism is still unclear.

The basolateral amygdala (BLA) has been implicated in mediating anxiety-related behaviors and processing nociceptive information and exhibits neuroplasticity in neuropathic pain [9]. The BLA contains neurons that preferentially respond to noxious stimuli [10]. Structural changes in the BLA region are most closely related to anxiety [11]. Studies have demonstrated that direct activation of cell bodies or their projections in the BLA is involved in both anxiogenic and socially aversive responses [12]. Evidence suggests that a stressful environment, elevated levels of stress hormones, and anxiety can lead to BLA hypertrophy [13, 14]. Recent work has also revealed that activating the excitatory pathway from the BLA relieves negative emotional behaviors, reducing anxiety and depression [15]. Although several studies have shown that the BLA is a key brain nucleus involved in chronic pain and negative emotion regulation, the neural mechanisms underlying changes in the BLA during anxiety-like behaviors induced by chronic pain are not well understood.

Dopamine (DA) is involved in descending pain modulation and behavioral responses to anxiogenic environmental stimuli [16, 17]. The BLA mainly receives input from glutamatergic neurons in the medial prefrontal cortex (mPFC) and input from dopaminergic neurons from the ventral tegmental area (VTA) [18], a nucleus that contains approximately 60% DA neurons [19]. DA receptors (DRs) are metabotropic G protein-coupled receptors that include two different classes: the D1 subfamily (DRD1 and DRD5) and the D2 subfamily (DRD2, DRD3, and DRD4) [20, 21]. Rodent studies have shown that noxious stimuli can affect the release of DA in the brain reward center [22]. Recent study results indicated that both DRD1 and DRD2 of the dentate gyrus (DG) are involved in acute pain modulation [23]. DRD2, but not DRD1, in the dorsolateral striatum, is involved in persistent pain modulation in a formalin test [24]. Intranucleus accumbens (NAc) infusion of a DRD1 agonist and DRD2 antagonist reduced stress-induced antinociceptive behaviors in persistent inflammatory pain [25]. However, a potential role of DRD1 and DRD2 in the BLA in neuropathic pain-induced anxiety has not been examined in the spared nerve injury (SNI) model.

Electroacupuncture (EA) is effective for relieving pain and emotional diseases [26, 27]. Moreover, it is widely used in China and all over the world because it is safer, more effective, and has fewer side effects than other treatments. Our previous studies have demonstrated that the effect of EA on complete Freund’s adjuvant (CFA)-induced pain aversion is associated with GAPDH, GLT-1, and PAK6 expression in the amygdala (AMY) [28]. However, it is still unclear whether EA plays a role in analgesia and anxiety relief through DRs in the BLA.

Based on the above findings, we aimed to explore whether the alleviation of allodynia and negative behavioral states by EA is associated with the modulation of the different DRs in the BLA in the SNI model of chronic neuropathic pain.

## Materials and Methods

### Animals and Methods

The male C57BL/6 mice (aged 8–10 weeks) used in all experiments were obtained from the Laboratory Animal Center of Zhejiang Chinese Medicine University, accredited by the Association for Assessment and Accreditation of Laboratory Animal Care (AAALAC). These mice were housed with 4–5 mice per cage, and each cage was covered with corn cob bedding. The mice were kept under standard environmental conditions at 23–25 °C (room temperature) and 40–60% humidity under a 12-h light–dark cycle (lights on at 8:00 am) with free access to food and water. Ventilation and air filtration systems were used during the study period. All animal care and experimental studies were approved by the Laboratory Animal Management and Welfare Ethical Review Committee of Zhejiang Chinese Medical University (permission number: ZSLL-2017–183).

### SNI Model Preparation

All SNI mice were anesthetized with isoflurane inhalation during the surgery. The skin on the left hind limb was exposed after it was shaved and disinfected with iodophor and alcohol. An incision was made, and the muscles underneath were opened by blunt dissection to expose the sciatic nerve, consisting of the sural, common peroneal, and tibial nerves. The sural and common peroneal branches were tightly ligated with nonabsorbent 6–0 sutures, and the nerves were transected. In addition, an approximately 2-mm section from the ligature was removed, while the tibial nerve was left intact. The muscle and skin were stitched layer-by-layer and disinfected with iodophor [29]. Sham mice received the same procedure, except that nerve ligation and severing were not carried out.

### EA Treatment

We selected the “Zusanli” (ST36) and “Sanyinjiao” (SP6) acupoints of the bilateral hind limbs for EA treatment. After the mice were immobilized, 0.16 × 7 mm acupuncture needles were inserted into the bilateral ST36 and SP6 acupoints to a depth of 5 mm. The two ipsilateral needles were connected with a HANS acupuncture point nerve stimulator (HANS-200A). The frequency was set at 100 Hz, the intensity was 0.3 mA, and the stimulation duration was 30 min. For the sham EA group, acupuncture needles were inserted into the bilateral ST36 and SP6 acupoints to a depth of 1 mm and connected to the electrodes without electrical stimulation. EA treatment was conducted 7 days, 9 days, 11 days, 13 days, and 15 days after SNI surgery. Mice in the other groups were immobilized in the same way but did not receive EA treatment.

### Von Frey Filament Test

Paw withdrawal thresholds (PWTs) were assessed using the von Frey filament test. Baseline stimuli values were obtained before SNI surgery. Mice were individually placed in a single transparent Plexiglas chamber on a wire mesh platform to test the middle of the left hind paw. The mice were allowed to acclimate for 30 min before the test. Then, the filament probe was inserted and focused at the middle of the surface of the left hind paw, and the pressure was gradually increased. Paw withdrawal, flinching, and licking of the claws were considered to be positive response behaviors. The interval of each measurement was more than 1 min. Each stimulus value was recorded, and the occurrence of three positive responses to five stimuli was defined as a PWTs. The mechanical pain threshold was repeatedly measured at baseline and 7 days and 14 days after SNI. All manipulations were performed by the same researcher.

### Elevated Plus Maze Test

Mice were transported to a behavioral testing room to habituate for at least 1 day prior to testing. The conditions of the behavioral testing room were as follows: dim lighting of 20 lx, a room temperature of 23–25 °C, a humidity of 45–55%, and noise at less than 40 dB. Behavioral tests were started 10–15 min after an injection of drugs or vehicle. The behavior of the mice during testing was recorded with a video tracking system (ANY-maze V6.14, Stoelting, USA).

The elevated plus maze (EPM) test is used to measure anxiety behaviors induced by open spaces and height [30]. On the 15th day after SNI surgery, the mice in all groups were subjected to EPM testing. The EPM consisted of two open arms (30 × 6 cm), two closed arms (30 × 6 × 15 cm), and a central area (6 × 6 cm), with the open and closed arms being perpendicular to form a cross. It was placed 35 cm above the floor in the behavioral testing room. Each animal was placed onto the center area with its head toward the open arm. After adapting to the experiment for 30 s, the mouse was allowed to explore the maze over a 5-min session. The apparatus was cleaned with 75% ethanol, and double-distilled water before the next experiment was performed.

### Open Field Test

Mice were transported to a behavioral testing room to habituate for at least 1 day prior to testing. The conditions of the behavioral testing room were as follows: dim lighting of 20 lx, a room temperature of 23–25 °C, a humidity of 45–55%, and noise at less than 40 dB. Behavioral tests were started 10-15 min after an injection of drugs or vehicle. The behavior of the mice during testing was recorded with a video tracking system (ANY-maze V6.14, Stoelting, USA).

The open field test (OFT) is used to assess locomotor activity and anxiety behaviors [31, 32]. All mice were subjected to the OFT 17 days after SNI surgery. The testing apparatus was a square area (40 × 40 × 40 cm) in an uncovered wooden case. The square floor was divided into 16 small areas, of which the center 4 areas made up the central zone, and the other areas made up the peripheral zone. Each mouse was gently placed in the central zone and allowed to freely explore the entire area for 5 min. After testing, the apparatus was cleaned similarly to the EPM.

### Western Blotting

Ninety minutes after behavioral testing, the mice were euthanized, and the brains were removed and stored in an ice boxer. The brain was cut into 0.5-mm-thick slices in continuous coronal sections using a mouse brain slice mold (RWD). The slices were placed on an ice block, and the bilateral AMY was then perforated and extracted using a blunt animal lumbar puncture needle (inner diameter, 1 mm). The obtained brain tissues were put into a 1.5-ml inlet EP tube, immediately placed into a liquid nitrogen tank, and transferred to a − 80 °C refrigerator for storage. After 80 µl of lysis buffer (RIPA and PMSF) and 2 small steel balls were added into the tubes with brain tissue, they were put into a homogenizer for grinding. The ground tissue was centrifuged at 4 °C at 15,000 RPM for 5 min to collect the supernatant. The protein concentration was determined using the BCA method. Protein samples (15 µg per lane) were electrophoresed on 10–12% SDS–polyacrylamide gels and transferred to 3 polyvinyl difluoride membranes. The membranes were blocked with 5% nonfat milk dissolved in TBST at room temperature for 1 h and incubated with the primary antibodies anti-DRD1 rabbit polyclonal antibody (1:1000, ab81296, Abcam) and anti-DRD2 rabbit polyclonal antibody (1:500, 55,084–1-AP, Proteintech) overnight at 4 °C. Secondary antibodies (1:5000, #7074, CST) were incubated with the membranes for 2 h at room temperature. Immunoreactivity was detected using enhanced chemiluminescence (Bio-Rad) and visualized with an ImageQuant LAS 4000 (EG). The density of each band was measured using ImageQuant TL 7.0 analysis software (GE). The bands were then eluted and incubated with HRP-conjugated anti-beta actin mouse monoclonal antibody (1:5000, #ab20272, Abcam).

### Drug Administration

The drugs used in the present study were as follows: the DRD1 agonist SKF38393 hydrobromide (Abcam, China, 0.3 µl per side), the DRD1 antagonist SCH23390 hydrochloride (Abcam, China, 0.3 µl per side), the DRD2 agonist quinpirole hydrochloride (Tocris, Bristol, UK, 0.8 µl per side), and the DRD2 antagonist sulpiride (Abcam, China, 0.4 µl per side). SKF38393 and SCH23390 were dissolved in 0.9% sterile saline at a concentration of 3 mg/ml. Quinpirole was dissolved in DMSO and then diluted with 0.9% sterile saline to the required volume at a concentration of 2.5 mg/ml. Sulpiride was dissolved in DMSO at a concentration of 3 mg/ml. The infusion rate for all drugs was 200 nl/min.

### Cannula Implantation and Microinjection

Mice were anesthetized with 0.3% pentobarbital sodium under aseptic conditions and fixed on a stereotaxic frame (RWD, 68025, Shenzhen, China). We bilaterally implanted guide cannulas 1 mm above the BLA in the brain (anteroposterior: − 1.17 mm; mediolateral: ± 3.2 mm; dorsoventral: − 4.0 mm) and used 1454 glue to fix the cannulas to the skull surface. Stainless steel screws were inserted into the skull, and dental cement was used to hold the screws and cannulas in place. An acupuncture needle was inserted into the catheter to the same length as the cannula to maintain its patency prior to microinjection. Cannula implantation was performed with maximal care to minimize infection, and the mice were housed in a clean cage and allowed 1 week of recovery before SNI surgery.

Before the microinjection procedure, the acupuncture needle in the cannula was pulled out. Bilateral microinfusions were carried out through internal cannulas that extended 1 mm beyond the tips of the guide cannulas to prevent blockage; the internal cannulas were connected to a 10-ml microsyringe mounted on the microinfusion pump. The infusion rate was 200 nl/min. The microsyringe remained in situ for an additional 3 min for drug diffusion after microinjection and was then removed slowly.

### Statistical Analysis

All data in the article are expressed as the mean ± standard error of the mean (SEM). An unpaired *t* test was used for comparisons between two groups. One-way ANOVA or two-way ANOVA followed by Tukey’s post hoc test was used for comparisons among ≥ 3 groups. The PWTs data were analyzed by two-way ANOVA followed by Tukey’s post hoc test. The EPM and OFT data were analyzed by one-way ANOVA followed by Tukey’s post hoc test. Statistical significance was indicated by *p* < 0.05 throughout.

## Results

### EA Relieves Mechanical Allodynia and Anxiety-Like Behaviors in SNI Mice

**Fig. 1:**
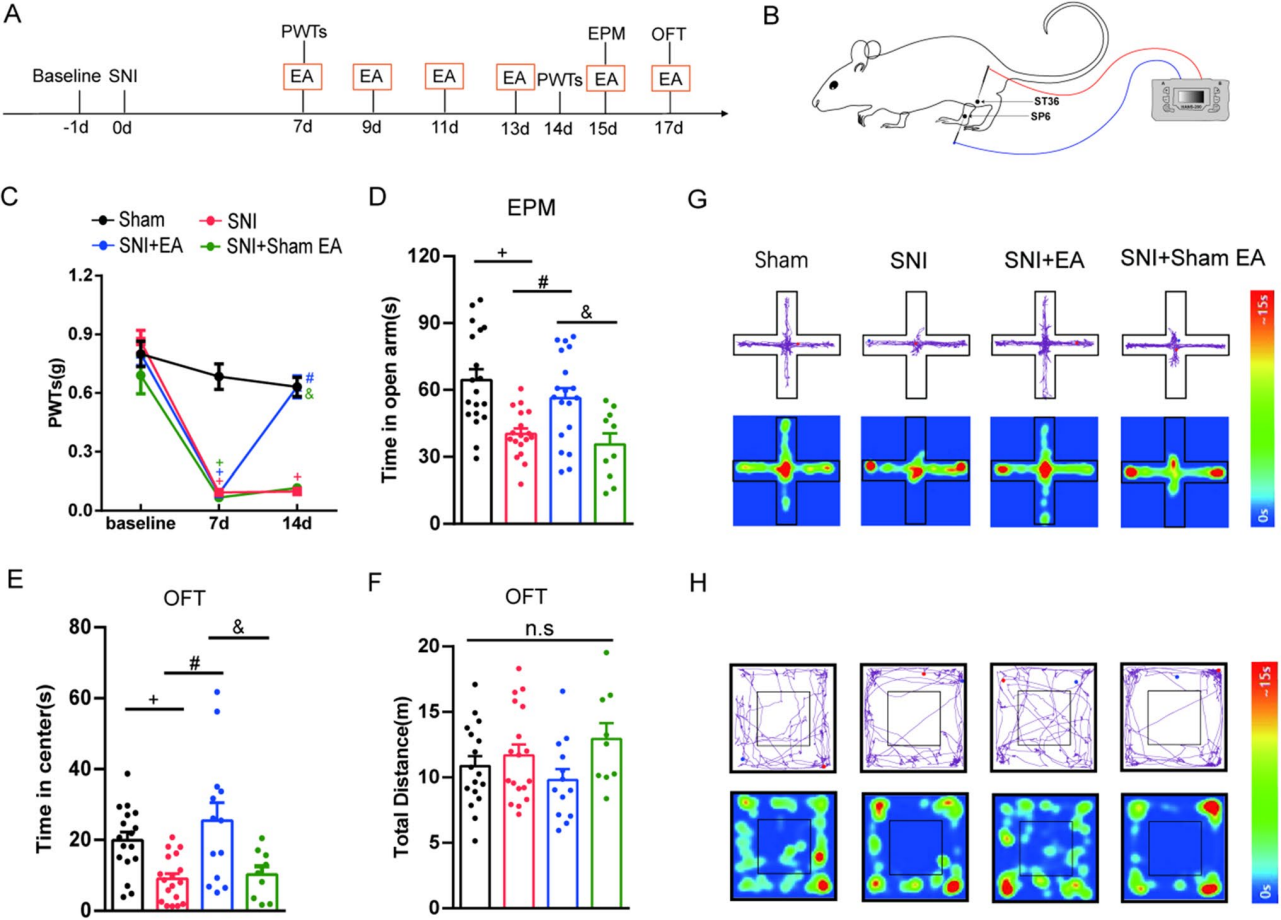
The effect of EA on the PWTs and anxiety-like behaviors in SNI mice. **A** Schematic of the experimental procedure. **B** Schematic of EA stimuli at acupoint sites ST36 and SP6 in SNI mice. **C** The effect of bilateral EA stimulation with 100 Hz on the PWTs. (*n* = 10–19 per group). **D**–**F** The effect of bilateral EA stimulation at 100 Hz on anxiety-like behaviors in the EPM (**D**) and OFT (**E**–**F**). (*n* = 9–19 per group). **G** Diagram showing representative movement and activity heatmaps in the EPM. **H** Diagram showing representative movement and activity heatmaps in the OFT. Data are expressed as the mean ± SEM. Tukey’s post hoc test: + *p* < 0.05 vs. the sham group; #*p* < 0.05 vs. the SNI group; &*p* < 0.05 vs. the SNI + sham EA group

First, we established an SNI model in which chronic neuropathic pain was induced. The PWTs of the left hind paw measured 1 day before SNI surgery were not significantly different among all four groups. At 7 days after surgery, the SNI mice showed significant mechanical allodynia in the ipsilateral hind paws, which lasted at least 14 days or longer. These results indicated successful establishment of the SNI model. EA treatment significantly increased the PWTs of SNI mice from days 7 to 14. However, SNI mice in the sham EA group did not exhibit increased PWTs (Fig. 1C). The anxiety-like behavioral responses of all groups were examined at 15 and 17 days after SNI surgery using the EPM and OFT, two tests that are commonly used to measure anxiety-related behaviors [33–35]. We found that the SNI mice spent a shorter time in the open arms of the EPM (Fig. 1D) and less time in the center area in the OFT (Fig. 1E) than the sham mice. The total distance traveled in the OFT was not significantly different among all groups (Fig. 1F). Mice stimulated with EA spent more time in the open arms in the EPM (Fig. 1D) and more time in the central area in the OFT (Fig. 1E). However, SNI mice in the sham EA group did not exhibit the alleviation of anxiety-like behaviors (Fig. 1D and E). Representative diagrams showing tracked movement and activity heatmaps each group in the EPM (Fig. 1G) and OFT (Fig. 1H) are shown. These data indicate that the SNI model of neuropathic pain induces mechanical allodynia and anxiety-like behaviors, both of which are effectively reduced by 100 Hz EA treatment.

### EA Treatment Reverses the Abnormal Expression of DRD1 and DRD2 in the AMY of SNI Mice

**Fig. 2:**
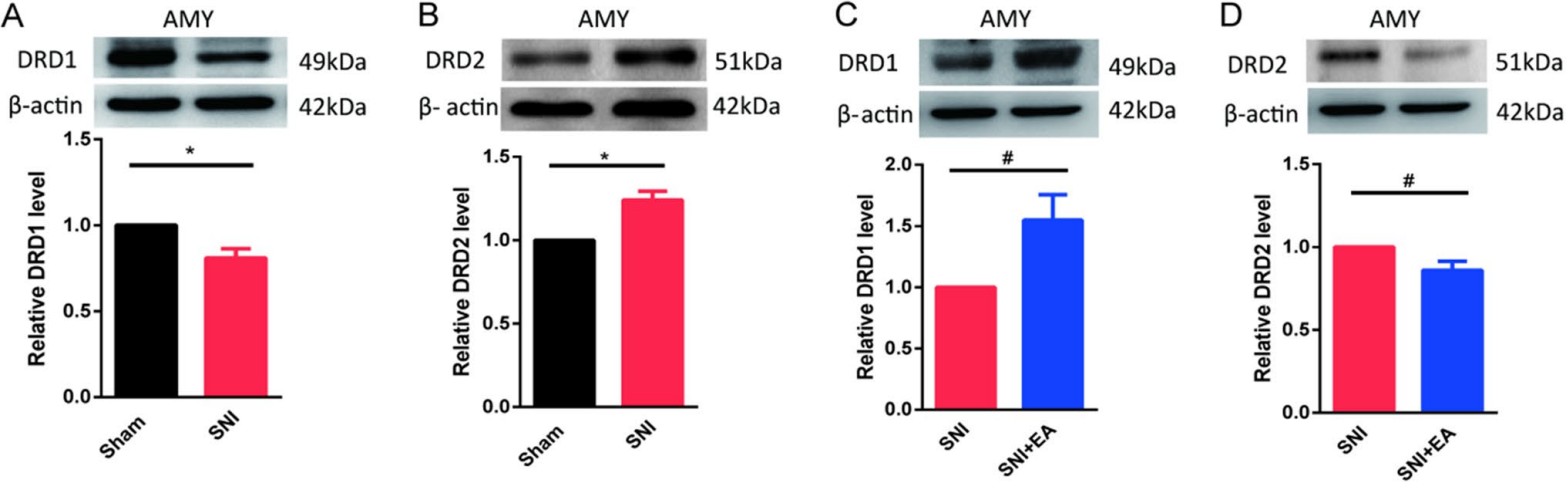
Expression of different DRs and EA-mediated regulation of these receptors in SNI mice. **A** and **B** Western blot images showing the protein levels and the quantification of DRD1 (**A**) and DRD2 (**B**) in the AMY 14 days after SNI surgery. (*n* = 5–6 per group). **C** and **D** Western blot images showing the protein levels and the quantification of DRD1 (**C**) and DRD2 (**D**) in the AMY of SNI mice treated with EA. (*n* = 6 per group). Data are expressed as the mean ± SEM. *p < 0.05 comparison of the two groups

The AMY is a key brain region associated with aversive emotions, including anxiety [36, 37]. We asked whether changes in the function of different DRs in the AMY were involved in SNI-induced anxiety-like behaviors. We used western blotting to detect the protein levels of DRD1 and DRD2 in the AMY of SNI mice. The protein levels of DRD1 (Fig. 2A) were significantly decreased, while the protein levels of DRD2 (Fig. 2B) were increased in the AMY 17 days after SNI surgery. Then, we detected the protein levels of DRD1 and DRD2 in the AMY after EA treatment. EA stimulation significantly increased DRD1 protein levels (Fig. 2C) but significantly decreased DRD2 protein levels (Fig. 2D) in the AMY compared to those in the SNI group.

### Inhibition of DRD1 in the BLA Contributes to Anxiety-Like Behaviors in Naive Mice, and EA Reverses this Effect

**Fig. 3:**
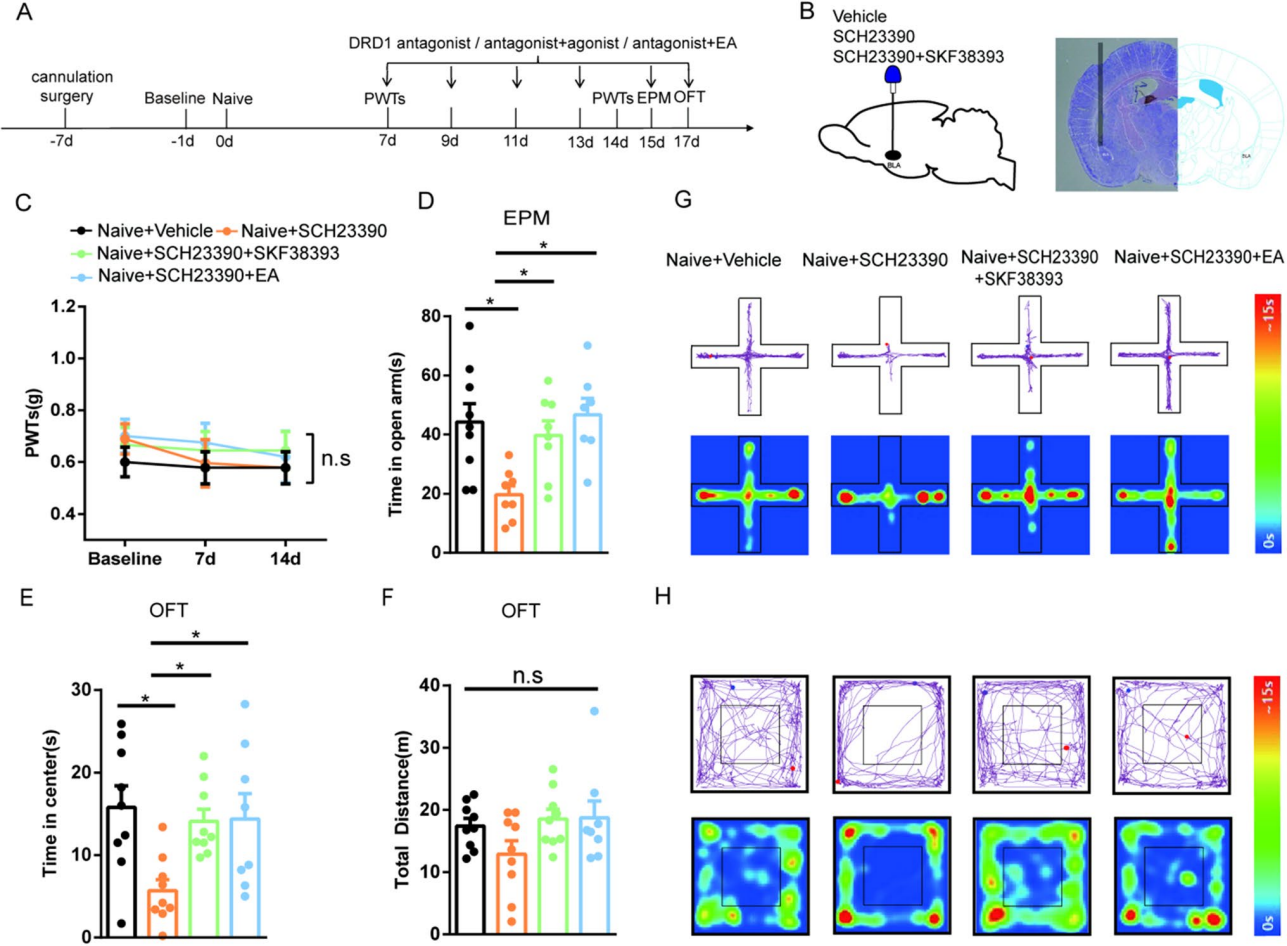
The effects of EA and the DRD1 agonist SKF38393 on anxiety-like behaviors induced by microinjection of the DRD1 antagonist SCH23390 in the BLA. **A** Schematic of EA treatment and drug injection and behavioral testing. **B** Diagram showing the cannulation location (left) and a representative image showing a coronal section with the cannula tip in the BLA from the injection of a mouse brain (right). **C** The effect of treatment in each group on the PWTs. (*n* = 8–9 per group). **D**–**F** The effect of treatment in each group on anxiety-like behaviors in the EPM (**D**) and OFT (**E**–**F**). (*n* = 7–9 per group). **G** Representative diagram showing tracked movement and activity heatmaps in the EPM. **H** Representative diagram showing tracked movement and activity heatmaps in the OFT. Data are expressed as the mean ± SEM. Tukey’s post hoc test: *p < 0.05 comparison of the two groups

Next, we first wondered whether the intra-BLA administration of the DRD1 antagonist SCH23390 in naive mice would affect PWTs and anxiety-like behaviors and whether EA would alleviate anxiety-like behaviors in a manner similar to that of the DRD1 agonist SKF38393 in naive mice. The experimental design is shown in Fig. 3A. A diagram of the cannulation location and a schematic representation of the injection of the cannula tip in the BLA in the mouse brain are shown in Fig. 3B. The PWTs of all groups were not altered on days 7 or 14 (Fig. 3C). Then, we measured anxiety-like behaviors by the EPM and OFT. We found that naive mice injected with SCH23390 in the BLA showed anxiety-like behaviors. SKF38393 treatment obviously relieved the anxiety-like behaviors induced by SCH23390 injection in naive mice. Moreover, compared with mice in the naive + SCH23390 group, the mice treated with EA spent more time in the open arms and central area in the behavioral tests (Fig. 3D and E). There was no difference in the total distance traveled in the OFT between the groups (Fig. 3F). Representative diagrams showing tracked movement and activity heatmaps of each group of mice in the EPM (Fig. 3G) and OFT (Fig. 3H) are provided. Taken together, these findings suggest that inhibition of DRD1 in the BLA contributes to anxiety-like behaviors in naive mice and that EA reverses this effect.

### EA Alleviates SNI-Induced Anxiety-Like Behaviors by Activating DRD1 in the BLA

**Fig. 4:**
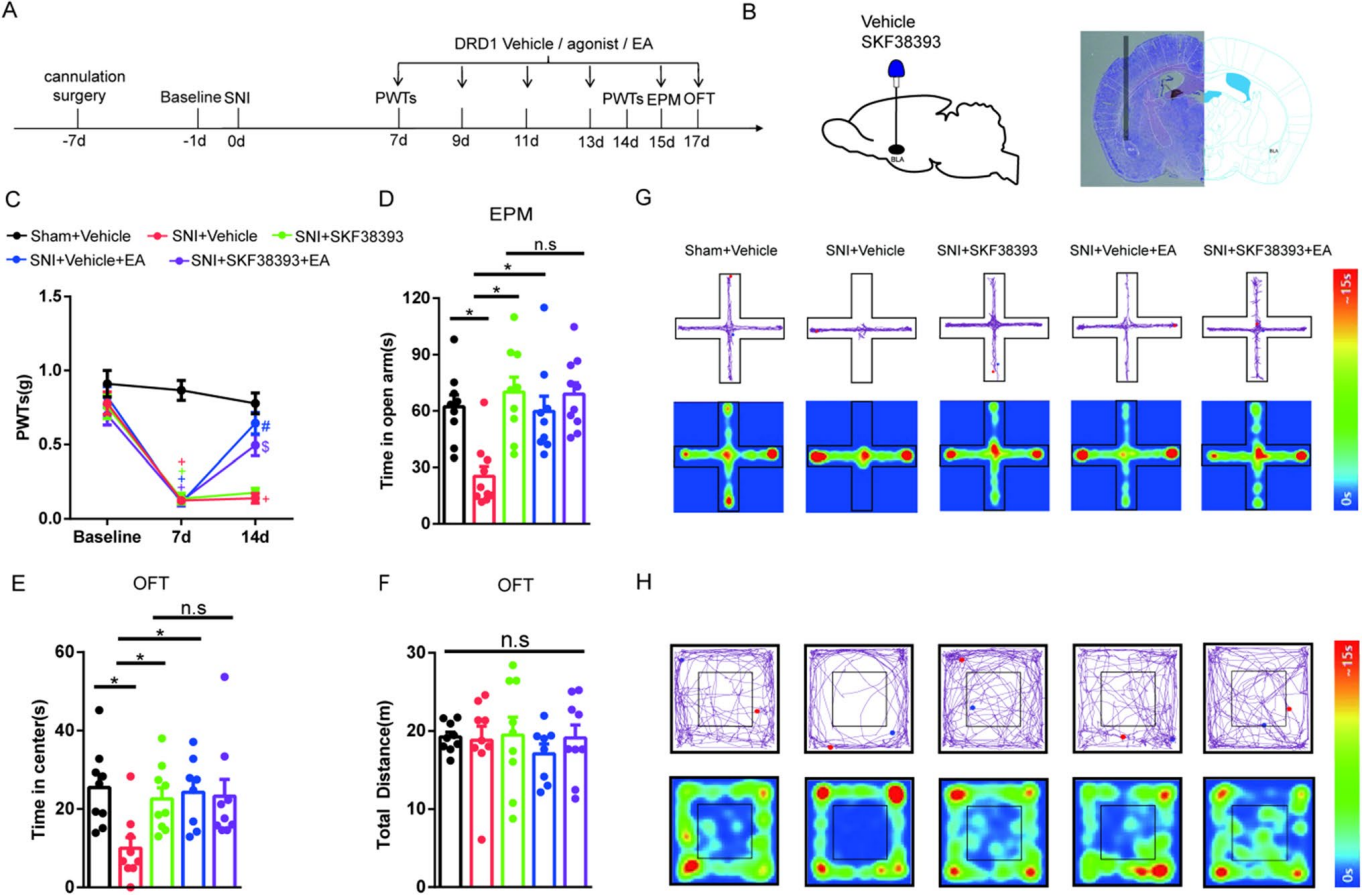
The effects of EA and the DRD1 agonist SKF38393 on pain-related allodynia and anxiety-like behaviors induced by SNI in mice. **A** Schematic of EA treatment and drug injection and behavioral testing. **B** Diagram showing the cannulation location (left) and a representative coronal section of the BLA from a mouse brain showing injection of the cannula tip (right). **C** The effect of treatment in each group on the PWTs. (*n* = 9–10 per group). **D**–**F** The effect of treatment in each group on anxiety-like behaviors in the EPM (**D**) and OFT (**E**–**F**). (*n* = 8–10 per group). **G** Representative diagram showing tracked movement and activity heatmaps from the EPM. **H** Representative diagram showing tracked movement and activity heatmaps from the OFT. Data are expressed as the mean ± SEM. Tukey’s post hoc test: ^+^*p* < 0.05 compared with the sham + vehicle group; ^#^*p* < 0.05 compared with the SNI + vehicle group; ^$^*p* < 0.05 compared with the SNI + SKF38393 group; *p < 0.05 comparison of two groups

DRD1 plays a critical role in regulating anxiety, as shown by previous studies [11, 38, 39]. We next determined that DRD1 in the BLA participates in alleviating hyperalgesia and anxiety-like behaviors induced by SNI in mice. The experimental design is shown in Fig. 4A. A diagram showing the cannulation location and a schematic representation of the cannula tip in the BLA during injection of a mouse brain are shown in Fig. 4B. As shown in Fig. 4C, in contrast to sham mice, SNI model mice display a significant reduction in PWTs on day 7 after modeling. On the 14th day after SNI surgery, the PWTs upon EA stimulation were significantly improved compared with those of SNI mice, but there was no change upon the injection of the DRD1 agonist SKF38393 in the mice. The PWTs of the group administered EA combined with SKF38393 treatment were higher than those of the group given intra-BLA administration of SKF38393. In addition, we found that SNI mice showed negative emotional behaviors on days 15 and 17 after modeling. SNI mice that received SKF38393 by intra-BLA injection or treatment with EA showed a significant increase in the time spent in the open arms and central area compared with SNI mice, but the total distance traveled in the OFT was not altered. There was no significant difference between mice administered EA combined with SKF38393 treatment and those treated with only SKF38393 (Fig. 4D–F). Representative diagrams showing tracked movement and activity heatmaps of each group of mice in the EPM (Fig. 4G) and OFT (Fig. 4H) are provided. Together, these results suggest that both the activation of DRD1 in the BLA and EA stimulation attenuate SNI-induced anxiety-like behaviors. EA stimulation relieves anxiety-like behaviors in SNI mice by activating DRD1 in the BLA.

### Activation of DRD2 in the BLA Contributes to Anxiety-Like Behaviors in Naive Mice, and EA Reverses this Effect

**Fig. 5:**
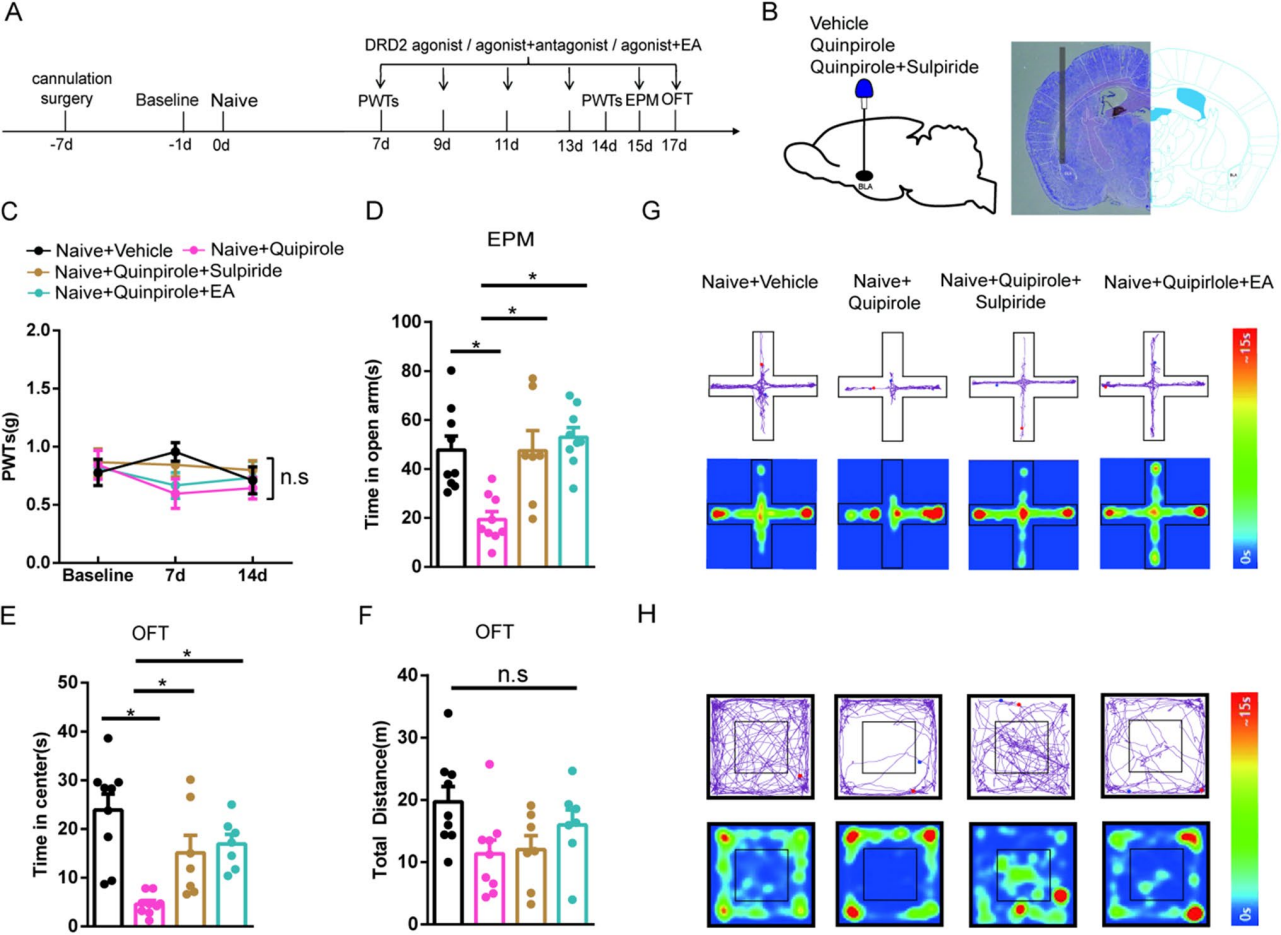
The effects of EA and the DRD2 antagonist sulpiride on anxiety-like behaviors induced by microinjection of the DRD2 agonist quinpirole in the BLA. **A** Schematic of EA treatment and drug injection and behavioral testing. **B** Diagram showing the cannulation location (left) and a representative coronal section of the BLA from a mouse brain showing the cannula tip upon injection (right). **C** The effect of treatment in each group on the PWTs. (*n* = 9 per group). **D**–**F** The effect of treatment in each group on anxiety-like behaviors in the EPM (**D**) and OFT (**E**–**F**). (*n* = 7–9 per group). **G** Representative diagram showing tracked movement and activity heatmaps from the EPM. **H** Representative diagram showing tracked movement and activity heatmaps from the OFT. Data are expressed as the mean ± SEM. Tukey’s post hoc test: *p < 0.05 comparison of the two groups

DRD2, a DR, has been reported to be involved in anxiety-like behaviors [16, 35, 39]. To verify the key role of DRD2 activity in the BLA in anxiety-like behaviors, we also used pharmacological methods to test this role in naive mice and examined the anxiety-like behaviors of each group. The experimental design is shown in Fig. 5A. A diagram showing the cannulation location and a schematic representation of the BLA from a mouse brain into which the cannula tip has been inserted are shown in Fig. 5B. We found that on days 7 and 14, the PWTs did not significantly differ between the groups (Fig. 5C). Naive mice injected with the DRD2 agonist quinpirole in the BLA exhibited anxiety-like behaviors on days 15 and 17, as the time spent in the open arms and central area was significantly decreased. After quinpirole and the DRD2 antagonist sulpiride were injected into naive mice, their anxiety-like behaviors were relieved to an extent equal to that of EA treatment combined with quinpirole treatment (Fig. 5D and E). Moreover, the total distance traveled did not differ between the groups (Fig. 5F). Representative diagrams showing tracked movement and activity heatmaps of the mice in the EPM (Fig. 5G) and OFT (Fig. 5H) are provided. These results indicate that activation of DRD2 in the BLA contributes to anxiety-like behaviors in naive mice and that EA reverses this effect.

### EA Alleviates SNI-Induced Anxiety-Like Behaviors by Inhibiting DRD2 in the BLA

**Fig. 6:**
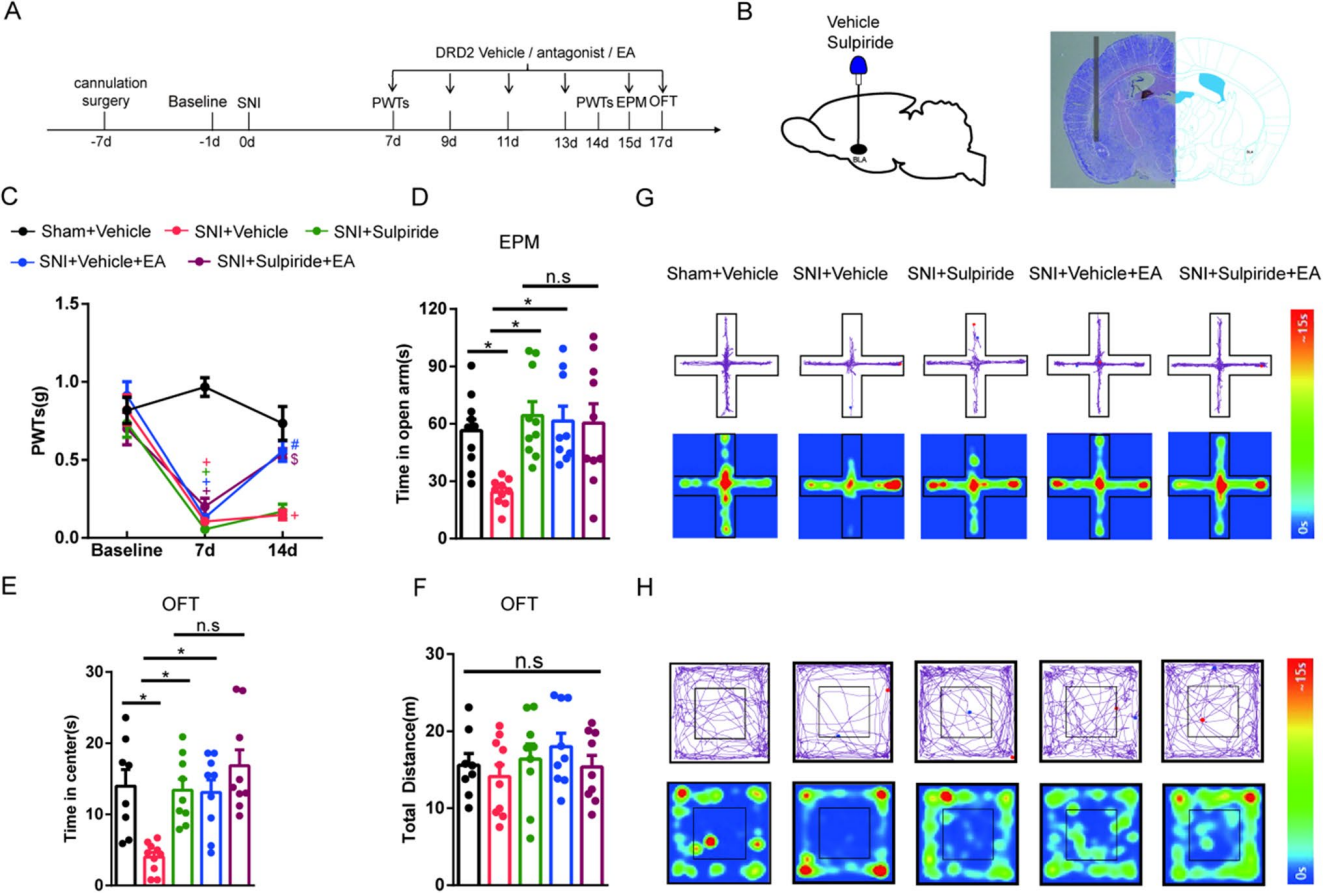
The effects of EA and the DRD2 antagonist sulpiride on pain-related allodynia and anxiety-like behaviors induced by SNI in mice. **A** Schematic of EA treatment and drug injection and behavioral testing. **B** Diagram showing the cannulation location (left) and a representative coronal section of the BLA from a mouse brain containing a cannula tip after injection (right). **C** The effect of treatment in each group on the PWTs. (*n* = 9–10 per group). **D**–**F** The effect of treatment in each group on anxiety-like behaviors in the EPM (**D**) and OFT (**E**–**F**). (*n* = 7–10 per group). **G** Representative diagram showing tracked movement and activity heatmaps from the EPM. **H** Representative diagram showing tracked movement and activity heatmaps from the OFT. Data are expressed as the mean ± SEM. Tukey’s post hoc test: ^+^*p* < 0.05 compared with the sham + vehicle group; ^#^*p* < 0.05 compared with the SNI + vehicle group; ^$^*p* < 0.05 compared with the SNI + sulpiride group; *p < 0.05 comparison between the two groups

Based on the above results, we speculated that the inhibition of DRD2 in the BLA may attenuate SNI-induced anxiety-like behaviors. Moreover, we speculated that EA relieved anxiety-like behaviors in SNI mice by antagonizing DRD2 in the BLA. The experimental design is shown in Fig. 6A. A diagram showing the cannulation location and a schematic representation of the BLA from a mouse brain with the cannula tip upon injection are shown in Fig. 6B. We found that 7 days after SNI surgery, the mice displayed a significant reduction in their PWTs compared with those of sham mice. Compared with mice in the SNI group, EA stimulation in mice significantly increased PWTs from days 7 to 14, but the DRD2 antagonist sulpiride did not relieve neuropathic pain in SNI mice. The PWTs of the group treated with EA combined with sulpiride were higher than those of the group given intra-BLA administration of sulpiride (Fig. 6C). Through the EPM and OFT, we found that SNI mice showed negative emotional behavior on days 15 and 17 after modeling. Furthermore, mice treated with EA and administered sulpiride spent more time in the open arms and central area in the behavioral tests than SNI mice. There was no significant difference between mice administered EA combined with sulpiride treatment and mice treated with only sulpiride (Fig. 6D and E). Moreover, the total distance traveled did not differ among the groups (Fig. 6F). Representative diagrams showing tracked movement and activity heatmaps from mice in the EPM (Fig. 6G) and OFT (Fig. 6H) are provided. Overall, these results suggest that both inhibiting DRD2 in the BLA and EA treatment attenuate SNI-induced anxiety-like behaviors. EA alleviates SNI-induced anxiety-like behaviors by inhibiting DRD2 in the BLA.

## Discussion

**Fig. 7:**
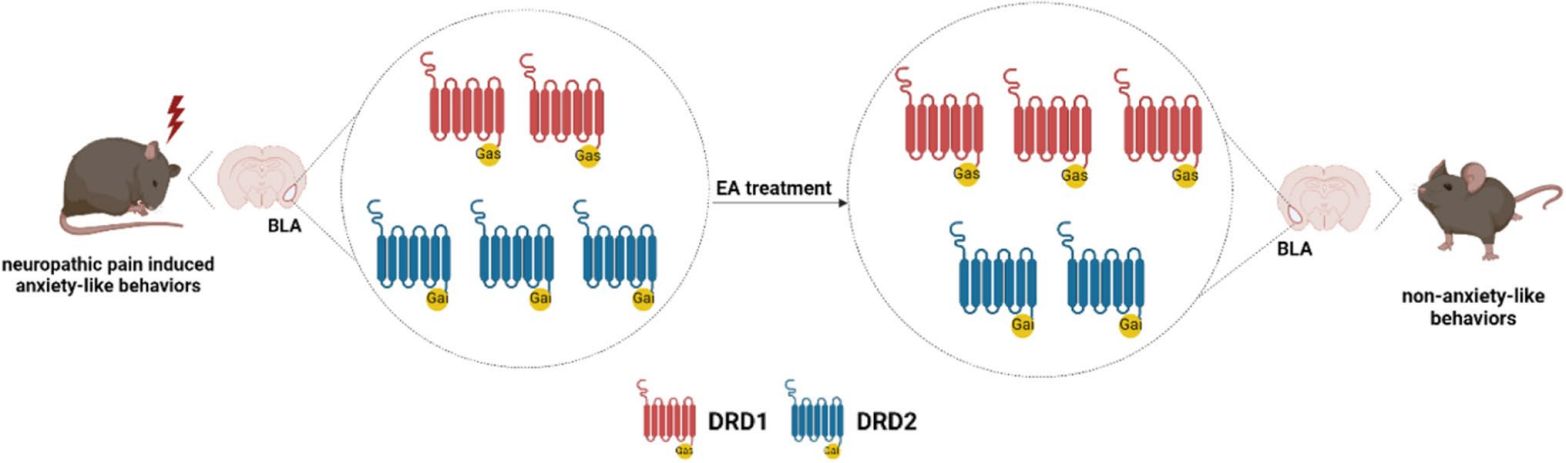
Proposed diagram of EA alleviates anxiety-like behaviors induced by chronic neuropathic pain via regulating different dopamine receptors of the BLA

In the present study, we demonstrated that EA could relieve neuropathic pain and anxiety-like behaviors induced by SNI in mice. We further investigated the potential mechanisms and found that the protein levels of DRD1 were significantly decreased and the protein levels of DRD2 were increased in the AMY 17 days after SNI surgery. Intra-BLA administration of DRD1 antagonist or DRD2 agonist in naive mice induced anxiety-like behaviors, with no change in PWTs. EA alleviated anxiety-like behaviors to an extent similar to that of the DRD1 agonist and DRD2 antagonist. Both activating DRD1 and antagonizing DRD2 in the BLA attenuated SNI-induced anxiety-like behaviors, but neither had an effect on mechanical allodynia. These results suggest that EA relieved anxiety-like behaviors in SNI mice by activating DRD1 or antagonizing DRD2 in the BLA. In addition, DRD1 and DRD2 in the BLA were not involved in the alleviation of SNI-induced neuropathic pain (Fig. 7).

The negative emotions induced by chronic pain are different from those induced by primary mental diseases. Its complex pathogenesis makes treatment very difficult and substantially affects quality of life. As a common analgesic method worldwide, EA affects multiple targets and acts on multiple levels, and EA has been widely used in the clinic and has been shown to achieve good clinical effects [40–42]. Accumulated evidence has found that EA can alleviate the abnormal pain sensation and negative emotions induced by chronic pain in rats [26, 27, 43]. We previously reported the analgesic and antidepressant effects of EA at different frequencies [44, 45]. We found that 100 Hz EA has a good analgesic effect on neuropathic pain and can also regulate negative emotions. The commonly used models of neuropathic pain include SNI [29], chronic constraint injury (CCI) [46], spinal nerve ligation (SNL) [47], and partial sciatic nerve ligation (PNL) [48]. The SNI model has proven to be robust, demonstrating substantial and prolonged changes in behavioral measures of mechanical sensitivity. These features closely mimic the cardinal symptoms of clinically described neuropathic pain disorders [49]. In this study, we induced anxiety-like behaviors and mechanical allodynia in mice through the SNI model and observed whether EA could treat these behaviors and mechanical allodynia. We found that the PWTs of mice were significantly decreased and that anxiety-like behaviors appeared at 15 and 17 days after SNI modeling. After EA treatment, the mechanical allodynia and anxiety-like behaviors of the SNI mice were alleviated, which suggested that EA had a therapeutic effect on mechanical allodynia and anxiety-like behaviors caused by SNI. Moreover, we also included a sham EA group, in which needs were inserted into acupoints ST36 and ST6 at a depth of 1 mm, but electrical stimulation was not applied. This is a common method used to establish the sham EA group [50–52]. In our study, we found that neither mechanical allodynia nor anxiety-like behaviors were relieved in SNI mice in the sham EA group. This suggests that electrical stimulation is necessary to alleviate mechanical pain and pain-related behaviors.

As part of the limbic system, the AMY, particularly the BLA region, is important in learning and processing positive emotions as well as negative emotions [10, 36, 53, 54]. BLA neurons project to many different downstream targets and receive neurons from different brain regions, so different projection targets may play different roles in mediating anxiety-related behaviors [55]. In a study of brain circuits, researchers found that the BLA-CeA pathway is a critical pathway for the regulation of anxiety, suggesting an anxiolytic effect of the excitatory BLA-CeA projection [15]. Another study found that optogenetic inhibition of BLA inputs to the vHPC reduced anxiety and that the activation of BLA-vHPC projections increased anxiety [55]. Therefore, BLA plays an increasingly significant role in regulating emotions. Moreover, both the presynaptic and postsynaptic phases of dopaminergic neurotransmission are impaired during chronic pain and in other emotional disorders [56, 57]. Recent evidence suggests that the inhibition of spinal DRD1/DRD2 could decrease the NMDAR-mediated activation of spinal neurons through Src kinase in a Gαq-protein-dependent manner to relieve chronic pain [58]. Previous studies have suggested that both DRD1 and DRD2 regulate anxiety, but they do so through different mechanisms. Furthermore, DRD1 and DRD2 in the anterior cingulate cortex (ACC) play opposite roles in trigeminal neuropathic pain [20]. Our study demonstrated that the protein levels of DRD1 were significantly decreased and those of DRD2 were increased in the AMY after SNI surgery, which suggested that DRD1 and DRD2 in the AMY have opposite effects on anxiety behaviors. EA stimulation significantly increased DRD1 and decreased DRD2 protein levels in the AMY compared to those in the SNI mice.

Based on the above results, we speculate that different DRs play different roles in physiological and pathological states. Therefore, we used pharmacological methods to verify the roles of DRD1 and DRD2 in different states. Our results showed that in the physiological state, antagonizing DRD1 in the BLA led to anxiety-like behaviors, which were alleviated by a DRD1 agonist or EA treatment in naive mice. These data showed that the anxiolytic effects of EA are similar to those of DRD1 activation in the BLA. However, activation or antagonism of DRD1 in the BLA had no effect on the PWTs, indicating that DRD1 in the BLA is not involved in mechanical pain. In the pathological state, not only activation of DRD1 in the BLA but also EA treatment could relieve anxiety-like behaviors induced by SNI in mice. Additionally, we found that the anxiolytic effects of EA are mediated by the activation of DRD1 in the BLA. EA stimulation can alleviate pain sensation but not through DRD1 in the BLA.

Then, we pharmacologically validated the role of DRD2 in the BLA and found that the activation of DRD2 in the BLA caused anxiety-like behaviors under physiological conditions, but the administration of a DRD2 antagonist or EA stimulation alleviated anxiety in naive mice. This finding suggested that the anxiolytic effects of EA are similar to those of BLA in antagonizing DRD2. However, neither activation nor antagonism of DRD2 in the BLA affected PWTs, indicating that DRD2 in the BLA is not involved in mechanical pain. In the pathological state, antagonization of DRD2 in the BLA or EA treatment alleviated SNI-induced anxiety-like behaviors, and EA inhibited anxiety-like behaviors by antagonizing DRD2 in the BLA. EA treatment can alleviate pain sensation, but this does not occur through DRD2 in the BLA.

Previous studies have demonstrated that pain-producing events generate hyperactivity in the BLA [10]. The anterior and posterior portions of the BLA are involved in controlling neuropathic pain [59]. Relevant studies have suggested that the BLA functions in pain modulation. Therefore, we speculate that DRD1 and DRD2 in the BLA are not involved in pain threshold modulation.

Acupuncture is an important part of Chinese medicine as well as a treatment for analgesia and relieving anxiety. The mechanism of how stimulating acupoints on the body surface affect the brain center has always been the focus of researchers. The team of Qiufu Ma found that EA drives sympathetic pathways in somatotopy- and intensity-dependent manners [60]. EA at the hindlimb ST36 acupoint can drive the vagal-adrenal anti-inflammatory axis in mice [61]. Therefore, we hypothesized that “Zusanli” (ST36) and “Sanyinjiao” (SP6) acupoints of the bilateral hind limbs may affect the dopamine system through the sympathetic and parasympathetic nervous system or the endocrine immune system. Research on how peripheral acupoints affect the central nervous system is also the core content of our research. Furthermore, we will focus on the mechanism by which leg acupoint stimuli affect the amygdala dopamine system.

## Conclusion

In conclusion, we have demonstrated a novel mechanism by which DRD1 and DRD2 in the BLA play different roles in neuropathic pain-induced anxiety-like behaviors through decreased DRD1 and increased DRD2. EA alleviates anxiety-like behaviors by activating DRD1 or antagonizing DRD2 in the BLA.

## Data Availability

All data are contained within the manuscript.

## Abbreviations

*DRD1*: Dopamine receptor D1
*DRD2*: Dopamine receptor D2
*BLA*: Basolateral amygdala
*SNI*: Spared nerve injury
*EA*: Electroacupuncture
*DA*: Dopamine
*AMY*: Amygdala
*PWTs*: Paw withdrawal thresholds
*EPM*: Elevated plus maze
*OFT*: Open field test
*DRs*: DA receptors
